# Systematic review: Outcome reporting bias is a problem in high impact factor neurology journals

**DOI:** 10.1371/journal.pone.0180986

**Published:** 2017-07-20

**Authors:** Benjamin Howard, Jared T. Scott, Mark Blubaugh, Brie Roepke, Caleb Scheckel, Matt Vassar

**Affiliations:** 1 College of Osteopathic Medicine, Oklahoma State University Center for Health Sciences, Tulsa, Oklahoma, United States of America; 2 Emergency Medicine, Oklahoma State University Medical Center, Tulsa, Oklahoma, United States of America; 3 Internal Medicine, Mayo Clinic, Scottsdale, Arizona, United States of America; 4 Department of Institutional Research, Oklahoma State University Center for Health Sciences, Tulsa, Oklahoma, United States of America; Cleveland Clinic, UNITED STATES

## Abstract

**Background:**

Selective outcome reporting is a significant methodological concern. Comparisons between the outcomes reported in clinical trial registrations and those later published allow investigators to understand the extent of selection bias among trialists. We examined the possibility of selective outcome reporting in randomized controlled trials (RCTs) published in neurology journals.

**Methods:**

We searched PubMed for randomized controlled trials from Jan 1, 2010 –Dec 31, 2015 published in the top 3 impact factor neurology journals. These articles were screened according to specific inclusion criteria. Each author individually extracted data from trials following a standardized protocol. A second author verified each extracted element and discrepancies were resolved. Consistency between registered and published outcomes was evaluated and correlations between discrepancies and funding, journal, and temporal trends were examined.

**Results:**

180 trials were included for analysis. 10 (6%) primary outcomes were demoted, 38 (21%) primary outcomes were omitted from the publication, and 61 (34%) unregistered primary outcomes were added to the published report. There were 18 (10%) cases of secondary outcomes being upgraded to primary outcomes in the publication, and there were 53 (29%) changes in timing of assessment. Of 82 (46%) major discrepancies with reported p-values, 54 (66.0%) favored publication of statistically significant results.

**Conclusion:**

Across trials, we found 180 major discrepancies. 66% of major discrepancies with a reported p-value (n = 82) favored statistically significant results. These results suggest a need within neurology to provide more consistent and timely registration of outcomes.

## Introduction

Bias plays a significant role in clinical research because it distorts the true effectiveness of interventions. One particular form of bias, known as outcome reporting bias, occurs when trialists fail to report prespecified outcomes, report primary outcomes that were not prespecified, report statistically significant secondary outcomes as primary outcomes, or report nonsignificant primary outcomes as secondary outcomes [1,2]. This form of bias is well documented in the medical literature [3,4,5,6,7,8,9]. Dwan et al. found that 40 to 62% of reviewed trials had at least one primary outcome that was changed, omitted, or newly introduced [10]. Because such reporting potentially affects clinical decision-making, growing interest exists for reviewing the medical literature, particularly randomized controlled trials (RCTs), for evidence of outcome reporting bias.

In medical research, evidence from RCTs forms the knowledge base on which physicians rely to guide clinical practice. Selective outcome reporting skews this knowledge base when only “positive” findings (i.e., statistically significant or showing a benefit) are reported in clinical trials [3]. Reports with positive findings are three times more likely to be published than trials with “negative” results. Outcome reporting bias has also been shown to favor statistically significant outcomes [11]. In a review of 42 meta-analyses with statistically significant results, eight (19%) became nonsignificant after adjusting for outcome bias and 11 (26%) overestimated the treatment effect by more than 20% [11]. In addition, less interest exists for publishing negative or inconclusive trials, and many registered trials (estimated at 53%) remain unpublished [12]. The nonpublication of negative results can subsequently lead to treatments being promoted even when the presumed benefits are actually unfavorable or harmful [13]. Further, even when negative results are reported, the level of detail may be inadequate; one study found that two-thirds of harmful outcomes in RCTs were incompletely reported [9]. The combination of selective reporting of positive trials and suppressed or inadequate reporting of negative trials can lead to expected treatment effects from a therapy exceeding those that are actually possible.

Many steps have been taken to decrease bias in clinical trials. In 1997, US federal law mandated a national registry for both federal and privately funded trials, leading to the creation of ClinicalTrials.gov [14]. This registry was of little use at first; however, since 2005, the International Committee of Medical Journal Editors (ICMJE) has required trial registration as a condition of publication [9]. This requirement increased the number of registered trials on ClinicalTrials.gov from 30 per week to 220 per week [6]. In 2007, the U.S. Food and Drug Administration (FDA) mandated trial registration before the enrollment of the first patient and required that all prespecified outcomes be clearly defined [14]. This explicit listing of outcomes facilitates comparisons between published outcomes and outcomes outlined in the registry, allowing for closer examination of outcome reporting bias.

The COMPARE Initiative, based at the Centre for Evidence-Based Medicine at the University of Oxford, compares registry-listed outcomes with published outcomes for all clinical trials published in the Journal of the American Medical Association, New England Journal of Medicine, Lancet, Annals of Internal Medicine, and The BMJ. For the period of October 2015–January 2016, COMPARE reviewed 67 trials for outcome reporting bias. Based on their findings, only nine trials had no discrepancies: 359 outcomes were not reported and 357 new outcomes were silently added. On average each trial reported only 58% of the prespecified trial outcomes and added 5.3 outcomes not prespecified in the trial registry [15]. Other groups, such as OPEN (Overcome failure to Publish nEgative fiNdings), the Cochrane Collaboration, and the AllTrials Initiative are also calling for greater transparency.

Although the requirements for registration, outcome reporting, and publication have yielded great improvements in the literature over the past two decades, analyses of published trials show overwhelming evidence of continued publication and outcome reporting bias across specialties including surgery, emergency medicine, anesthesia, dermatology, oncology, and internal medicine [3,4,5,6,7,8,9]. However, little is known about the prevalence of outcome reporting bias in the neurology literature. The aim of this study was to investigate selective outcome reporting bias of RCTs in the three highest impact neurology journals from 2011 to 2015.

## Methods

The primary goal of this study was to assess potential discrepancies between the primary and secondary outcomes in registered RCTs and the associated reports published in high impact factor neurology journals. Secondary outcomes were to highlight whether outcome reporting discrepancies favor statistically significant outcomes, whether there was any correlation between funding source and likelihood of outcome reporting bias, and whether any temporal trends in outcome reporting bias occurred during the time examined. We also catalogued any incidental findings during data extraction and analysis that warranted further examination. To accomplish these aims, we performed a methodological systematic review of the three highest impact factor neurology journals from 2011 to 2015. This study did not meet the regulatory definition of human subjects research according to 45 CFR 46.102(d) and (f) of the Department of Health and Human Services’ Code of Federal Regulations [16] and was not subject to Institutional Review Board oversight. Li et al. [17], the Cochrane Handbook for Systematic Reviews of Interventions[18], and the National Academies of Science, Engineering, and Medicine’s (previously the Institute of Medicine) Standards for Systematic Reviews [19] were consulted to ensure best practices regarding data extraction and management. PRISMA guideline [20] items 1, 3, 5–11, 13, 16–18, and 24–27 were applied to ensure reporting quality for systematic reviews in addition to SAMPL guidelines [21] for reporting descriptive statistics. Prior to initiation of the study, we registered it with the University hospital Medical Information Network Clinical Trials Registry (UMIN-CTR) with registry number: R000025976 UMIN000022541. All extracted data for this study are publicly available on figshare (https://figshare.com/articles/Selective_Reporting_Bias_in_Neurology_Project_date_06_01_16-07_01_16/3799503).

### Eligibility criteria for studies for this review

We searched for RCT indexed in PubMed between January 1, 2011, and December 31, 2015. This time period was selected because it is several years after the mandatory ICMJE trial registration policy and allowed enough time to observe reporting trends in neurology journals. RCTs published in the following journals were included: The Lancet: Neurology, Neurology, and Annals of Neurology. These journals were selected based their top 3 rankings in the 5-year impact factor of Journal Citation Reports. The National Institutes of Health definition of clinical trial—“a research study in which one or more human subjects are prospectively assigned to one or more interventions (which may include placebo or other control) to evaluate the effects of those interventions on health-related biomedical or behavioral outcomes” [22]—was used for this study. For our purposes, prospective assignment had to occur by random assignment of a participant to a condition. We included RCTs, follow-up studies on previously performed RCTs that analyzed different primary outcomes at a later time point, and RCTs that used a crossover method. The following study types were excluded from the study: meta-analyses, observational studies (including cohort, case—control, and cross sectional), ongoing studies, letters to the editor, commentary or discussion pieces, articles with only a title or lacking an abstract, studies examining a mechanism, animal/in vitro studies, and simulation-based studies.

### Search strategy for identifying relevant studies

With the assistance of a medical research librarian, a PubMed search was performed of the three neurology journals by limiting articles to “randomized controlled trials” between the previously detailed dates. By nature of the journals, all articles were published in English. The final search was deployed as follows: ("Ann Neurol"[Journal] OR "Lancet Neurol"[Journal]) OR "Neurology"[Journal] AND ((Clinical Trial[ptyp] OR Clinical Trial, Phase I[ptyp] OR Clinical Trial, Phase II[ptyp] OR Clinical Trial, Phase III[ptyp] OR Clinical Trial, Phase IV[ptyp] OR Randomized Controlled Trial[ptyp]) AND ("2011/01/01"[PDAT]: "2015/12/31"[PDAT])).The search took place on May 20, 2016.

### Study selection and data extraction

Citations obtained during the search were uploaded into Endnote X7.5. Two investigators (B.H. and J.S.) independently screened the abstract and title of each citation for possible study inclusion after completing an internally developed training platform. Any disagreement about potential inclusion was resolved by a consensus meeting. Resolution of difficult cases by a third party (M.V.) was planned but was not ultimately needed. Those citations excluded from the study were copied into an Excel spreadsheet, and each was coded for reason of exclusion. Investigators were blinded to registration status (whether the trial had been registered in a clinical trial registry) during screening to minimize observer bias.

After initial screening, the citations were imported into the Agency for Healthcare Research and Quality’s Systematic Review Data Repository (SRDR) [23] for data extraction. For internal calibration and to prevent discrepancies in extraction, each investigator underwent SRDR and data extraction training. Investigators would first view training videos produced by the AHRQ on navigating SRDR, creating an extraction form, and entering data (http://srdr.training.ahrq.gov/). Investigators next performed an AHRQ training exercise composed of creating an extraction form and using it to enter data from one study. After completing AHRQ’s training modules, investigators undertook a second set of training exercises, consisting of an internally developed training video series that explained this study’s SRDR extraction form and data entry procedures. After all training modules had been completed, investigators extracted data from three clinical trials from unrelated medical specialties, using this study’s data extraction form, and compared extractions to an answer key developed by a third investigator (C.S.)

Two investigators (B.H. and J.S.) independently reviewed the full-text articles for each study and extracted data using SRDR. At least once per day, these investigators would trade articles and repeat the other’s data extraction. This procedure allowed them to cross-validate each other’s work and to improve the accuracy and efficiency of data extraction. Any disagreements were resolved by discussion between the pair. A third-party reviewer (M.V.) was available for further adjudication but was not needed. We extracted the following items from the published RCTs: primary outcome(s), secondary outcome(s), date of subject enrollment, trial registry database and registration number, timing of assessment in primary outcomes (e.g., pain at 12 hours, mortality at 6 months), sample size, any discrepancies between publication and registry disclosed by the author in the publication, and funding source. For the purpose of our study we classified funding source into the following categories: (1) private (e.g., Mayo Clinic or philanthropic), (2) public (government or public university laboratory), (3) industry/corporate (e.g., GlaxoSmithKline), (4) university hospital, (5) mixed, or (6) undisclosed. For RCTs that reported multiple primary and secondary outcomes, we recorded each explicitly stated outcome. If a primary outcome was not explicitly stated as such in the publication, the outcome stated in the sample size estimation was used. If no outcome was explicitly stated in the text or in the sample size calculation, the article was excluded from the study. When sample size was not explicitly stated in the article, we used the “number randomized.” If authors failed to differentiate between primary and secondary outcomes in the publication, these non-delineated outcomes were coded as “unable to assess” and excluded from comparison.

The clinical trial registry or registration number was obtained from each published RCT, if stated, during full-text review/data extraction. If a registration number was listed in the RCT without a trial registry, a search was made of ClinicalTrials.gov, the International Standard Randomized Controlled Trial Number Register (ISRCTNR), the World Health Organization’s (WHO’s) International Clinical Trial Registry Platform (ICTRP), and any country-specific clinical trial registry identified in the publication. The following characteristics were used to match registered study to publication: title, author(s), keyword, country of origin, sponsoring organization, description of study intervention, projected sample size, and dates of enrollment. When a publication did not explicitly state information regarding registration of a study, the authors were contacted via email using a standardized email template and asked about registration status. If after 1 week there was no reply, a second email was sent. If there was no reply from authors 1 week after the second email, the study was considered to be unregistered.

Each registered study was located within its respective registry and data was extracted individually by 2 independent investigators (B.H. and J.S.). Prior to registry data extraction, both investigators underwent trial registry training including training videos on how to perform searches and access the history of changes in ClinicalTrials.gov and the WHO trial registry, a tutorial video about locating desired content from trial registry entry, and access to a list of all WHO-approved trial registries. Each investigator also had to successfully complete a sample data extraction from an unrelated study registry entry. The following data were extracted using a standardized form on SRDR: date of trial registration, date range of subject enrollment, original primary registered outcome(s), final primary registered outcome(s), date of initial primary outcome registration, secondary registered outcome(s), sample size if listed, and funding source, if disclosed, using previously defined categories. Although registration quality was not the focus of this study, registered trials lacking a clearly stated primary outcome and timing of assessment were excluded from consideration. Studies that were found to be registered after the end of subject enrollment were excluded from the study because of the inability to adequately assess outcome reporting bias.

To be approved by the WHO, a trial registry must meet ICMJE criteria, including documentation of when changes are made to that particular study’s registry entries. If an included study employed this feature, we recorded both the primary outcome from time of initial registration as well as the primary outcome listed in the final version in the registry entry. Departing from the methods of previous authors in this field of research, we did not exclude studies in WHO-approved registries that did not time-stamp the date of initial primary outcome registration. Per the International Standards for Clinical Trial Registries section 2.4 [24], WHO-approved registries are required to time-stamp registry-approved changes to any registered trial including data additions, deletions, and revisions. Therefore, if a WHO-approved trial registry did not display a history of changes, we recorded the date the registry application was approved as the date of initial primary outcome registration. Additionally, the listed primary outcome was recorded as both the initial registered and final registered primary outcome. In non—WHO-approved trial registries, if a date of initial primary outcome registration was not listed, this trial was excluded from our study.

Investigators (B.H., J.S., and M.V.) then compared the primary outcomes listed in the publication to the initial registered primary outcomes for consistency. Decisions were made by consensus. Outcomes were deemed consistent if every primary outcome detailed in the publication was listed as such in the registry, and vice versa. We defined five major discrepancies according to the classification system described by Chan et al. [25] and refined by Mathieu et al. [6]:

1. The registered primary outcome was reported as a secondary outcome in the published article.
2. The registered primary outcome was omitted in the published article.
3. A new primary outcome was introduced in the published article (i.e., a registered secondary outcome became primary in the article, or an outcome omitted in the registry was introduced as primary in the article).
4. The published primary outcome was described as a secondary outcome in the registry.
5. The timing of assessment of the registered and published primary outcomes differed.

Additionally, because trial registries allow authors to update their primary outcomes at any point, we also looked for matches between original registered primary outcomes and published primary outcomes. In a case in which the original registered primary outcome did not match the published primary outcome and changes were made after submission of the article, the study was flagged as having a discrepancy. Cases in which additional clarifying information about existing outcomes were added but no change was made to the registered primary outcome were not identified as having a discrepancy. Finally, we made note if an outcome was categorized as primary or secondary in the registry but was left unspecified in the publication. These instances were not recorded as being an upgrade or a downgrade, but the irregularity was recorded.

Articles with discrepancies that were found using the system of Mathieu et al. [6] were also assessed to determine if discrepancies favored statistically significant results. As with Mathieu et al. [6], a discrepancy was considered to favor statistically significant results if an outcome was statistically significant and described as a primary outcome in the publication despite not being defined as a primary outcome in the registry, or when a registered primary outcome was statistically insignificant and omitted or defined as a nonprimary outcome in the published article.

Data were initially exported and analyzed using Excel 2013. Google Documents was utilized as a data comparison platform.

## Results

**Fig 1:**
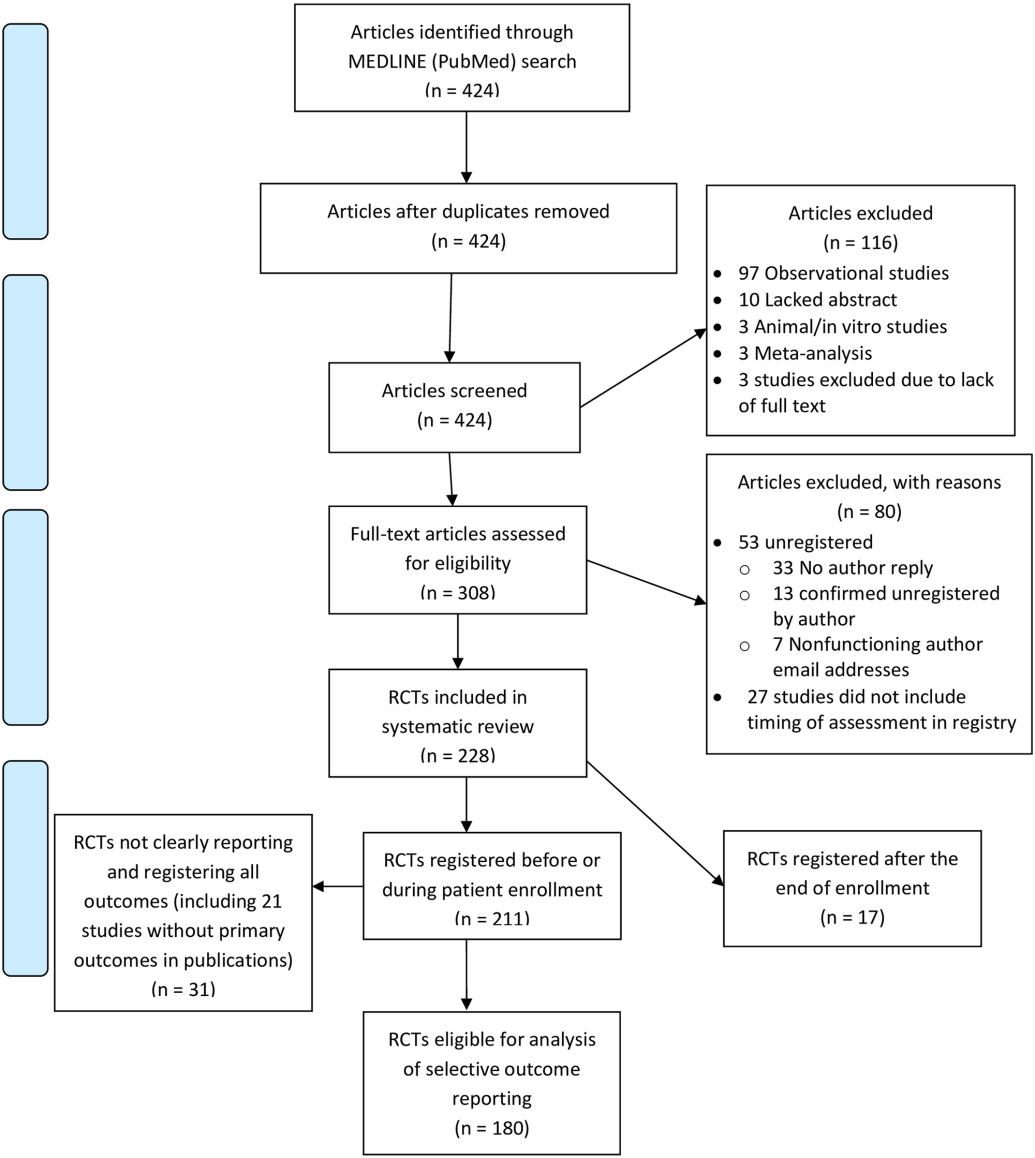
Prisma diagram of search strategy.

Our initial search yielded 424 articles. Two hundred twenty-eight RCTs were included, and of these, 211 were registered prior to patient enrollment. RCTs for which a primary or secondary outcome was not mentioned in their publication or RCTs that did not define a primary outcome in their registry were excluded, leaving us with a final sample size of 180 for data analysis. Other exclusion criteria are detailed in the Prisma diagram (Fig 1).

Out of the RCTs that were included in our analysis, 81 (45%) were registered during patient enrollment. The remaining 99 (55%) were prospectively registered. Seventeen studies were retrospectively registered and excluded from our analysis per exclusion criteria. For studies that did not explicitly state their enrollment dates, we used the study start and end dates that were included in the registry.

Table 1 details the demographics of the RCTs included in our study. Six different registry databases were included in our study. More than half (n = 158, 88%) of the RCTs that we analyzed were registered with ClinicalTrials.gov. The next most common database that our trials were registered with was ISRCTN (n = 11, 6%), followed by EudraCT (n = 4, 2%), Netherlands Trial Registry (n = 4, 2%), Australian New Zealand Clinical Trials Registry (n = 2, 1%), and the Dutch Trial Registry (n = 1, 0.6%).

**Table 1:** Characteristics of randomized controlled trials published in the three highest-impact journals between January 1, 2010 and December 31, 2015.

n = 154	
Journal	
* Neurology*	86
* Lancet Neurology*	82
* Annals of Neurology*	12
Funding Source	
Industry/Corporate	71
Public	60
Private	21
Mixed	11
None Disclosed	1
Registry	
Australian New Zealand Clinical Trials Registry	2
ClinicalTrials.gov	158
EudraCT	4
ISRCTN	11
Netherlands Trial Registry	4
Dutch Trial Registry	1
Time of Registration	
Registered before or during patient enrollment	163
Retrospectively registered	17
Publication Year	
2011	26
2012	37
2013	28
2014	49
2015	38
2016	2
Number of Patients Enrolled	
<100	72
100–199	32
200–499	36
>500	40

Our primary outcome was to evaluate the frequency of major outcome discrepancies present in high impact factor neurology journals. Our sample of 180 RCTs included 180 major discrepancies. Of these 180 major discrepancies, 10 (6%) primary outcomes were demoted, 38 (21%) primary outcomes were omitted from publication, 61 (34%) unregistered primary outcomes were added to the publication, 18 (10%) secondary outcomes were upgraded to primary in the publication, and 53 (29%) outcomes had changes to the timing of assessment.

Industry-funded RCTs composed the majority of our sample, but these trials contained fewer major discrepancies than most funding sources (32/71, 45%) (Table 2). Privately funded RCTs contained the most major discrepancies (16/21, 76%), followed by publicly funded RCTs (32/60, 53%). RCTs with mixed funding made up 26 of the 180 RCTs and contained the fewest discrepancies (11/26, 42%). RCTs that did not disclose funding information accounted for 2 of the 180 RCTs and contained one discrepancy (50%).

**Table 2:** Published RCTs that were registered before or during trial completion and have major discrepancies with their trial registries, and the effect of these discrepancies on the statistical significance of published outcomes, by funding source.

	Total		Private		Public		Industry		Mixed		None Disclosed	
No. of published RCTs	180		21		60		71		26		2	
No. of published RCTs with major discrepancies between registry and publication	92	(51%)	16	(76%)	32	(53%)	32	(45%)	11	(42%)	1	(50%)
No. of major discrepancies between registry and publication	180		29		68		67		15.0		1	
Registered primary outcomes demoted in publication	10	(6%)	4	(14%)	3	(4%)	2	(3%)	1	(7%)	0	(0%)
Registered primary outcomes omitted from publication	38	(21%)	3	(10%)	17	(25%)	15	(22%)	3	(20%)	0	(0%)
Unregistered primary outcomes added to publication	61	(34%)	9	(31%)	25	(37%)	25	(37%)	2	(13%)	0	(0%)
Registered secondary outcomes promoted in publication	18	(10%)	4	(14%)	5	(7%)	7	(10%)	2	(13%)	0	(0%)
Timing of assessment of primary outcomes differs	53	(29%)	9	(31%)	18	(26%)	18	(27%)	7	(47%)	1	(100%)
No. of major discrepancies between registry and publication	180		29		68		67		15		1	
No reported p-values	7	(4%)	2	(7%)	1	(1%)	4	(6%)	0	(0%)	0	(0%)
Reported p-values	82	(46%)	15	(52%)	32	(47%)	30	(45%)	5	(33%)	0	(0%)
Major discrepancy favors statistical significance	54	(66%)	9	(60%)	19	(59%)	24	(80%)	2	(40%)	0	(0%)
Major discrepancy does not favor statistical significance	34	(41%)	6	(40%)	13	(41%)	6	(20%)	3	(60%)	0	(0%)
No. of RCTs containing major discrepancies favoring statistical significance	38		7		13		15		3		0	

Of 82 major discrepancies with reported p-values, 54 (66%) outcomes were changed in favor of statistically significant results. Industry-funded RCTs were more likely to have major discrepancies favoring statistical significant results, followed by RCTs with public, private, and mixed finding. There were four mixed-funded RCTs with evaluable discrepancies. None of the RCTs with undisclosed funding had any major discrepancies that were evaluable.

We also examined major discrepancies by journal (Table 3). The Annals of Neurology had the highest frequency of major discrepancies (9/12, 75%), followed by Neurology (52/86, 60%). The Lancet Neurology had the smallest proportion of major discrepancies (29/82, 35%). Of the major discrepancies with reported p-values, Neurology had the highest frequency favoring statistically significant results (38/56, 68%), followed by Lancet Neurology (14/21, 67%), and Annals of Neurology (3/5, 60%). A temporal trend was not apparent in the frequency of major discrepancies and the year the RCT was conducted (Table 4).

**Table 3:** Published RCTs that were registered before or during trial completion and have major discrepancies with their trial registries and the effect of these discrepancies on the statistical significance of published outcomes, by funding source.

	Total		*Annals of Neurology*		*Lancet Neurology*		*Neurology*	
Number of published RCTs	180		12		82		86	
Number of published RCTs with major discrepancies between registry and publication	87	(48%)	9	(75%)	29	(35%)	52	(60%)
Number of major discrepancies between registry and publication	180		19		55		106	
Registered primary outcomes demoted in publication	10	(6%)	4	(21%)	2	(4%)	4	(4%)
Registered primary outcomes omitted from publication	38	(21%)	5	(26%)	16	(29%)	17	(16%)
Unregistered primary outcomes added to publication	61	(34%)	2	(11%)	18	(33%)	41	(39%)
Registered secondary outcomes promoted in publication	18	(10%)	1	(5%)	4	(7%)	13	(12%)
Timing of assessment of primary outcomes differs	53	(29%)	7	(37%)	15	(27%)	31	(29%)
Number of major discrepancies between registry and publication	180		19		55		106	
Did not report p-values	7	(4%)	2	(11%)	3	(5%)	2	(2%)
Reported p-values	82	(46%)	5	(26%)	21	(38%)	56	(53%)
Major discrepancy favors statistical significance	52	(63%)	3	(60%)	14	(67%)	38	(68%)
Major discrepancy does not favor statistical significance	33	(40%)	2	(40%)	7	(33%)	18	(32%)
Number of RCTs containing major discrepancies favoring statistical significance	38		3		10		25	

**Table 4:** Year-by-year frequency of major discrepancies between published and registered outcomes in RCTs registered before or during patient enrollment (n = 181), and the effect of these discrepancies on the statistical significance of published outcomes, by year.

Publication year	No. of major discrepancies	No. of evaluable discrepancies,		No. of evaluable discrepancies whose discrepancies favor statistically significant results	
2011	25	14	56%	7	50%
2012	42	19	45%	16	84%
2013	20	10	50%	3	30%
2014	62	25	40%	19	76%
2015	27	13	48%	7	54%
2016	4	2	50%	0	0%
Total	180	82	46%	52	63%

## Discussion

Our results indicate that trial registration and selective outcome reporting are problems that need to be addressed in neurology literature. Overall, only about 40% of trials were prospectively registered. The remaining 60% were improperly registered: they were either registered during patient enrollment or after study completion. Furthermore, we found evidence of 180 outcome inconsistencies across 180 RCTs. In many cases, these inconsistencies favored changes in accordance with statistically significant results. Given that outcome inconsistencies may have significant implications for clinical practice, solutions should be implemented to directly address this problem.

First, it appears that journal policies may have a limited effect. Annals of Neurology, for example, states the following in its Instructions for Authors (as of August 7, 2016): “All clinical trials must be registered in a database that meets the requirements set forth by the ICMJE: (1) The registry must be publicly accessible. (2) It must be open to all registrants and managed by a not-for-profit group. (3) The registry must have a mechanism to guarantee accuracy and validity of the information submitted” and further states that “endpoints in the paper should be those in the registration. If other endpoints are used, this should be pointed out and the reasoning discussed in the text” [26]. Of the journals included in our analysis, Annals of Neurology contained the largest percentage of trials with major discrepancies, yet it provides the most explicit requirements for trialists regarding alterations to outcomes. Likewise, Neurology’s Instructions for Authors (as of August 7, 2016) state, “Neurology requires investigators to register their clinical trials in a public trials registry and to provide the identification of the clinical trial registry and the clinical trial identification number… Neurology will not consider retrospectively registered trials for publication.” [27]. The journal also requires trialists to provide a statement including the trial’s registry and the clinical trial identifier number. Again, given the nature of our findings, these policies do not appear to be strictly enforced. Finally, Lancet Neurology’s Authors Instructions state (as of August 7, 2016), “We require the registration of all interventional trials, whether early or late phase, in a primary register that participates in WHO's International Clinical Trial Registry Platform… We also encourage full public disclosure of the minimum 20-item trial registration dataset at the time of registration and before recruitment of the first participant.” [28]. The 20-item data set includes explicitly listing the primary and secondary outcomes. Given the volume of research being submitted and published on an annual basis, practical considerations or limited resources may limit the extent to which journals can actually monitor these problems.

Some journals are taking action to improve outcome reporting [29]. Both The BMJ and BMJ Open now require a declaration of transparency from the primary author. This declaration states, “the lead author affirms that this manuscript is an honest, accurate, and transparent account of the study being reported; that no important aspects of the study have been omitted; and that any discrepancies from the study as planned (and, if relevant, registered) have been explained” [30]. Another proposal would require authors to submit the trial registry information and an explanation of any deviation between information in the registry and the submitted manuscript as a precondition for publication [31]. These additional steps may promote greater adherence to using prespecified outcomes and encourage more transparent practices.

Second, clinical trialists should improve their research practices beginning with study registration. It is outside the scope of this study to evaluate the motives of trialists or comment on whether outcome discrepancies were intended to be deceptive or simply represent a lack of awareness by trialists. But despite these assumptions, trialists need to be informed about the bias being introduced from outcome switching and the consequences associated with these alterations. ClinicalTrials.gov released a statement for public comment that, among other things, clarifies the definitions of primary, secondary, and tertiary outcomes since these definitions were too vague in the FDA Amendments Act (the legislation requiring trial registration prior to patient enrollment). Greater clarity regarding the registration of outcomes may play a role in reducing selective outcome reporting among trialists.

Third, peer reviewers may affect selective outcome reporting in both negative and positive ways. One study examined the use of registry information during the peer review process [32]. Among respondents, 34.3% used information from the trial registry during peer review of the trial. A large majority of these reviewers either reported discrepancies in their comments or advised editors not to accept the manuscript for publication. Peer reviewers who did not use registry information cited the lack of a trial registration number as a primary reason for not including this information during the review. It seems prudent that all parties should work toward common solutions to limit this significant form of bias in clinical trials.

In summary, we identified many cases of selective outcome reporting in the neurology literature. This finding is discouraging given that the journals we included for review have explicit policies regarding trial registration and outcome consistency between registration and publication. A further discouraging realization was that while we found many cases of reporting bias in the highest impact factor neurology journals, this practice might be running more rampantly in lower impact factor journals. Parties should work toward adopting better practices, beginning with the trialist. Peer reviewers and journal editors should more consistently monitor these practices so that trials without justified outcome changes will not go to publication. There were limitations to this study, however we consider these to be minor and to have had a minimal impact on the results. One limitation was that studies that did not specify the scale used to assess the primary outcome in the publication but mentioned it in the clinical trial registry were considered discrepant due to our inability to accurately ascertain that they used the same intended scale of measurement throughout their study. Additionally, studies that did not mention how they were going to measure or assess their primary outcomes in a clear manner that could easily be interpreted were considered discrepant as well. Despite these limitations, it is clear that selective outcome reporting in neurology literature is prevalent and stricter practices should be adopted to ensure quality reporting.

## Supporting information

S1 Checklist Prisma checklist. (DOC)
